# Risks of Aromatase Inhibitor-Related Cardiotoxicity in Patients with Breast Cancer in Asia

**DOI:** 10.3390/cancers14030508

**Published:** 2022-01-20

**Authors:** Wei-Ting Chang, Po-Wei Chen, Hui-Wen Lin, Yu-Hsuan Kuo, Sheng-Hsiang Lin, Yi-Heng Li

**Affiliations:** 1 Division of Cardiology, Department of Internal Medicine, Chi-Mei Medical Center, Tainan 710402, Taiwan; cmcvecho2@gmail.com; 2 Institute of Clinical Medicine, College of Medicine, National Cheng Kung University, Tainan 701401, Taiwan; huntershobow@gmail.com; 3 Department of Biotechnology, Southern Taiwan University of Science and Technology, Tainan 710301, Taiwan; 4 Department of Internal Medicine, National Cheng Kung University Hospital, College of Medicine, National Cheng Kung University, Tainan 701401, Taiwan; alice882233@gmail.com; 5 Biostatistics Consulting Center, National Cheng Kung University Hospital, College of Medicine, National Cheng Kung University, Tainan 701401, Taiwan; 6 Division of Oncology, Department of Internal Medicine, Chi-Mei Medical Center, Tainan 710402, Taiwan; beethovan@gmail.com; 7 Department of Public Health, College of Medicine, National Cheng Kung University, Tainan 701401, Taiwan

**Keywords:** breast cancer, aromatase inhibitors, SERMs, mortality, MACCEs, age, cancer stage

## Abstract

**Simple Summary:**

In Asian breast cancer patients, whether the risks of major adverse cardio- and cerebrovascular events (MACCEs) are different between users of aromatase inhibitors (AIs) and selective estrogen receptor modulators (SERMs) remains uncertain. In this nationwide cohort study, the risks of MACCEs were significantly higher in users of SERMs compared with users of AIs in those who were at an old age (above 50 years old) or with advanced cancer stage (stage 3–4). Although the choice of hormone therapies is primarily based on the effectiveness regarding cancer survival, AI treatment should be considered for patients for whom the benefits outweigh the risks.

**Abstract:**

Background: Despite a preferred endocrine therapy for women with estrogen and progesterone receptor-positive breast cancer, aromatase inhibitors (AIs) have been reported to increase risks of cardiovascular events. Given that breast cancer patients in Asia are younger at diagnosis, it is urgent to investigate this safety concern. Methods: Through the Taiwanese National Cohort, we identified breast cancer patients initiating selective estrogen receptor modulators (SERMs) or AIs from 2010 to 2016. Outcomes includes major adverse cardio- and cerebrovascular events (MACCEs). The average follow-up duration was five years. Results: We identified 16,730 breast cancer patients treated with SERMs and 11,728 receiving AIs. The population was older and had more comorbidities in the AI group than in the SERM group. After adjusting for age, cancer stage, cancer therapies, cardiovascular drugs and comorbidities, despite similar risks of MACCEs between AI and SERM users, the risk of HF was significantly higher in patients treated with SERMs after adjusted mortality as a competing risk. When divided by the age of 50 years, despite a similar MACCEs in the younger population, MACCEs remained significantly higher in the older population who received SERMs. Conclusions: In this Asian cohort, we found that among patients of old age or with advanced cancer stage, the use of SERMs was associated with a higher risk of cardiovascular events than the use of AIs.

## 1. Introduction

With the improvement of anticancer therapies, the number of long-term cancer survivors of breast cancer has increased, but survivors face threats of cardiovascular complications, including heart failure (HF) and thromboembolic events [1,2]. Patients with tumors that are estrogen receptor (ER)- and progesterone receptor (PR)-positive are eligible for hormone therapy, including selective estrogen receptor modulators (SERMs) and aromatase inhibitors (AIs) [3,4]. Compared with SERMs, AIs have become the preferred adjuvant treatment for postmenopausal women given favorable clinical outcomes [3,5,6]. Emerging evidence reports a potential risk of major adverse cardio- and cerebrovascular events (MACCEs) with AI use compared with tamoxifen, a most frequently used SERM, use [6,7,8,9]. Nevertheless, there are inconsistent findings between randomized controlled trials and observational studies [6,9]. Conversely, patients, especially the elders, treated with tamoxifen were at a higher risk for venous thrombotic events during the first 2 years after exposure. Despite some plausible theories indicating an association between AIs and hyperlipidemia, the biological mechanism of AI-induced cardiotoxicity remains largely unknown [10,11]. Additionally, no definitive guidelines specifically suggest the choice of hormone therapies between AIs and SERMs regarding the potential cardiotoxicity in patients with breast cancer [12].

To date, the number of patients diagnosed with breast cancer in Asia has continuously increased [13,14,15]. In contrast to Western patients with breast cancer, Asian patients are relatively younger at diagnosis [14,15,16]. This implies that most patients with breast cancer in Asia are diagnosed before menopause and have few cardiovascular risk factors [3]. Most importantly, the majority of previous studies focused on breast cancer patients in the Western world, while only a very limited number of Asian populations were included [13,15]. Therefore, it is crucial to evaluate the incidences and clinical characteristics of AI- and SERM-induced cardiotoxicity in Asia. Herein, using the Taiwan National database, we aimed to compare cardiovascular safety between SERMs and AIs in Asian populations.

## 2. Patients and Methods

### 2.1. Study Design

Through Taiwanese National Health Insurance Research Database (NHIRD) and National Cancer Registry, we identified breast cancer patients initiating hormonal therapy with either SERMs or AIs from 2010 to 2016. The data used in this study were the original claims database for reimbursement of all Taiwanese residents from the NHIRD [17,18]. The cohort dataset included age, sex, medications, procedures and all medical diagnoses. To protect patients’ privacy, all data were anonymized or pseudonymized. The diagnosis codes in the NHIRD were identified using the International Classification of Diseases, Ninth Revision, Clinical Modification (ICD-9-CM) for cases before 2015 and the International Classification of Diseases, Tenth Revision, Clinical Modification (ICD-10-CM) for cases since 2016. It is also feasible to link and continuously follow up all of the claim’s data belonging to the same patient within the NHIRD. This study was approved by our institutional review committee (IRB A-EX-109-021; CV code: 10406-E01) and they granted a waiver of informed consent due to its retrospective nature. We included women who received hormone therapies at least twice within the 365 days after the index date of first diagnosis and had no shift of medications. The exclusion criteria for this study were a history of breast cancer (registry for catastrophic illness patients diagnosed before 2010), previous exposure to SERMs (tamoxifen, toremifene, fulvestrant), gonadotropin-Releasing hormone analogue (goserelin and leuprorelin) or AIs (exemestane, anastrazole and letrazole), incomplete medical records, age less than 18 years, and nonfemale sex. Patients who reached outcomes of mortality or MACCEs were also excluded. In addition, we identified patients with breast cancer of all stages using the nationwide cancer registration system in Taiwan. All comorbid conditions and corresponding treatments starting a year prior to diagnosis were extracted from the National Health Insurance Research Database, as well as medication records of breast cancer diagnosis and treatments. The ICD diagnosis and treatment codes were used to identify concomitant medical diseases, medications and procedures (Table S1). Information on age, sex, medical history, concomitant medications within the previous three months, and medications or procedures used during the index admission were captured from the database. The flow chart of this study was displayed in Figure S1.

### 2.2. Study Endpoint

The primary outcome was a composite endpoint of MACCE, which included new-onset acute myocardial infarction (AMI), HF, and ischemic stroke (including transient ischemic attack). All patients were followed up from the index date to death or loss to follow-up. Because ICD-9 cm was replaced by ICD-10 cm by the Taiwan National Health Insurance in 2016, both ICD 9 and 10 codes (Supplementary Table S1) were used to identify endpoints in the primary outcome during the follow-up. The median follow-up duration were five years.

### 2.3. Statistical Analysis

Continuous variables are presented as the means ± standard deviations, and categorical variables are presented as numbers and percentages. Because of the nonrandomized nature of the study, propensity score analysis was performed to minimize any selection bias caused by differences in the clinical characteristics between groups. The propensity score is defined as the probability of exposure to the treatment conditional on a study subject’s baseline characteristics. In this study, the propensity score for receiving either SERMs or AIs was computed using multivariate logistic regression analysis, conditional on the covariates including index year, age, cancer stage, anti-cancer therapies (bilateral ovariectomy, anthracyclines, taxanes, 5-fluorouracil, cyclophosphamide), CV medication, including angiotensin- converting enzyme inhibitors/angiotensin receptor blockers (ACEI/ARB), beta blocker, statins, anti-platelet agents, anti-coagulants, digoxin, mineralocorticoid receptor antagonist (MRA), comorbidities, including coronary artery disease, peripheral artery disease, hypertension, diabetes mellitus, hyperlipidemia, valve disease, chronic obstructive pulmonary disease, asthma, atrial fibrillation, chronic kidney disease and end stage renal disease (ESRD). Distributions of the clinical characteristics in the 2 groups were evaluated with the absolute standardized mean difference (ASMD) rather than statistical testing. ASMD was calculated as the mean or proportion of a variable divided by the pooled estimate of the standard deviation of that variable, and an ASMD <0.1 indicates a negligible difference between the two groups. A multivariate Cox proportional hazards model with inverse probability of treatment weighting (IPTW) was then used to examine the relationship between the endpoints and different treatments. The same variables used for multivariate logistic regression analysis after propensity score matching were also used in the multivariate Cox model. The HRs and their 95% CIs were calculated from the Cox models after adjusting for all of these potential confounders. In addition, considering the mortality effects that may reduce the incidence of events, the competing risk approach (subdistribution HR; sHR) was also used to estimate the risk of MACCEs from the Cox regression model after adjusting for all of these potential confounders. A Kaplan–Meier curve was constructed for the primary outcome of MACCE and new-onset HF, and the log-rank test was used to compare the difference between groups. We used the same Cox proportional hazards model to estimate P values for interactions in the subgroup analysis. SAS 9.4 for Windows (SAS Institute Inc., Cary, NC, USA) was used for all data analyses.

## 3. Results

### 3.1. Demographic Characteristics of Breast Cancer Patients Receiving SERMs or AIs

Using the NHIRD from 2010 to 2016, we identified 116152 patients newly diagnosed with breast cancer. Among them, 16,730 patients were treated with SERMs, while 11,728 received AIs (Table 1). The population was older in the AI group than in the SERM group (62.53 ± 9.05 y/o vs. 49.63 ± 11.55 y/o). In terms of cancer stage and treatment, more patients who received AIs were at an advanced cancer stage (stage III or IV) than those who received SERMS (19.8% vs. 6.07%), while more SERM users were treated with anthracyclines (23.93%), and AI users were treated with taxanes (30.98%). The ratio of bilateral ovariectomy was similar between these two groups (AIs vs. SERMs as 0.63% vs. 1.13%). Notably, compared with patients who received SERMs, those receiving AIs had more comorbidities, including hypertension, diabetes, hyperlipidemia and coronary artery disease, and they received more cardiovascular drugs, such as ACEIs/ARBs, statins and antiplatelet and anticoagulant agents.

### 3.2. Risks of MACCEs between Breast Cancer Patients Receiving SERMs or AIs

The crude risks of MACCEs, including HF, AMI and ischemic stroke, were significantly higher among SERM users than among AI users (Table 2). With regard to older ages and more coexisting medical conditions among AI users, ages, cancer stage, cancer therapies, cardiovascular drugs and comorbidities were adjusted. Interestingly, after adjustment, although the differences in the risks of AMI and ischemic stroke diminished between groups, the risk of HF was persistently higher in patients receiving AIs than in patients receiving SERMs (adjusted HR: 0.884; CI: 0.810–0.966, *p* = 0.007). Furthermore, given a high risk of death among cancer patients, we adjusted the HR with mortality as a competing event, while the sHR of HF remained significantly reduced in AI users compared with SERM users (sHR: 0.885; CI: 0.812–0.966, *p* = 0.006).

**Figure 1:**
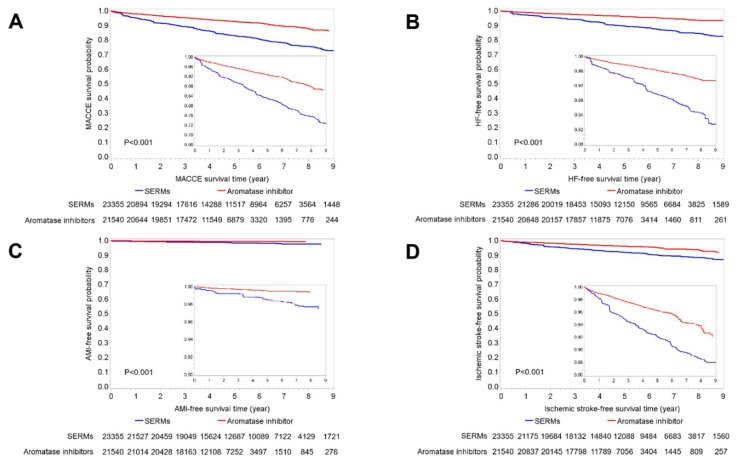
The estimated probabilities of patients being free from (**A**) major adverse cardio-cerebral events (MACCE), (**B**) heart failure (HF) (**C**) acute myocardial infarction (AMI) and (**D**) ischemic stroke among breast cancer patients receiving either selective estrogen receptor modulators (SERMs) or aromatase inhibitors (AIs).

In the five-year follow-up period, the rates free from MACCEs were also lower among AI users than among SERM users (Figure 1A). After one, three and five years from the index date, the rates free from MACCEs were 95.15%, 89.25% and 83.08%, respectively, among SERM users, compared with 97.96%, 95.29% and 92.76% in AI users. With regard to the different cardiovascular outcomes, the rates of HF (Figure 1B), AMI (Figure 1C) and ischemic stroke (Figure 1D) were persistently higher among patients receiving SERMs than among patients treated with AIs.

### 3.3. Focusing on Breast Cancer Patients at Different Ages

Given that, to date, the use of AIs is approved only for patients at menopause [1], we further aimed to investigate the effects of AI use on cardiovascular outcomes among patients at different ages. When divided by the age of 50 years old, we found that surprisingly, among young patients (<50 y/o) who received AIs, the risks of MACCEs were similar between AI and SERM users (adjusted HR: 0.982; CI: 0.699–1.381, *p* = 0.919) (Table 3). After adjusting the HR with mortality as a competing event, the risk of MACCEs remained insignificantly different between groups (sHR: 0.976; CI: 0.693–1.372, *p* = 0.887). In contrast, when focusing on patients aged above 50 y/o, the risk of MACCEs remained lower among AI users compared with SERM users (adjusted HR: 0.798; CI: 0.746–0.854, *p* < 0.001). In terms of each cardiovascular endpoint, there were significant reductions in the risks of HF (adjusted HR: 0.741; CI: 0.677–0.812, *p* < 0.001), AMI (adjusted HR: 0.749; CI: 0.578–0.971, *p* = 0.029) and ischemic stroke (adjusted HR: 0.877; CI: 0.797–0.965, *p* = 0.007) among AI users compared with SERM users. The abovementioned risks were persistently lower among AI users after adjusting for mortality as a competing risk.

### 3.4. Focusing on Breast Cancer Patients with Different Cancer Stages

According to emerging evidence, the benefits of hormone therapy in reducing the risk of breast cancer recurrence and progression seem to be independent of cancer stage [1]. Nevertheless, whether the effects of AI on cardiovascular outcomes are associated with cancer stages remains uncertain. We further divided the study patients into relatively early (stage 0–2) and advanced cancer stages (stage 3–4) and observed that in patients with early cancer stages, the risks of MACCEs (adjusted HR: 0.930; CI: 0.862–1.002, *p* = 0.058) were insignificantly different between AI and SERM users (Table 4). Despite a significant risk reduction in AMI among AI users (adjusted HR: 0.54; CI: 0.397–0.735, *p* < 0.001), the phenomenon diminished in terms of the outcomes of HF (adjusted HR: 0.910; CI: 0.821–1.009, *p* = 0.074) and ischemic stroke (adjusted HR: 0.968; CI: 0.873–1.073, *p* = 0.537) compared with the risks among SERM users. In contrast, focusing on patients with advanced cancer stage, there were significantly reduced risks of MACCEs (adjusted HR: 0.492; CI: 0.425–0.569, *p* < 0.001) among AI users compared with SERM users. Similarly, the risks of HF (adjusted HR: 0.470; CI: 0.391–0565, *p* < 0.001) and ischemic stroke (adjusted HR: 0.434; CI: 0.337–0.559, *p* < 0.001) were also lower among patients treated with AIs than among those treated with SERMs, but in contrast, the risk of AMI increased (adjusted HR: 2.567; CI: 1.366–4.827, *p* = 0.003).

### 3.5. Subgroup Analysis of Hormone Therapy-Related MACCEs

**Figure 2:**
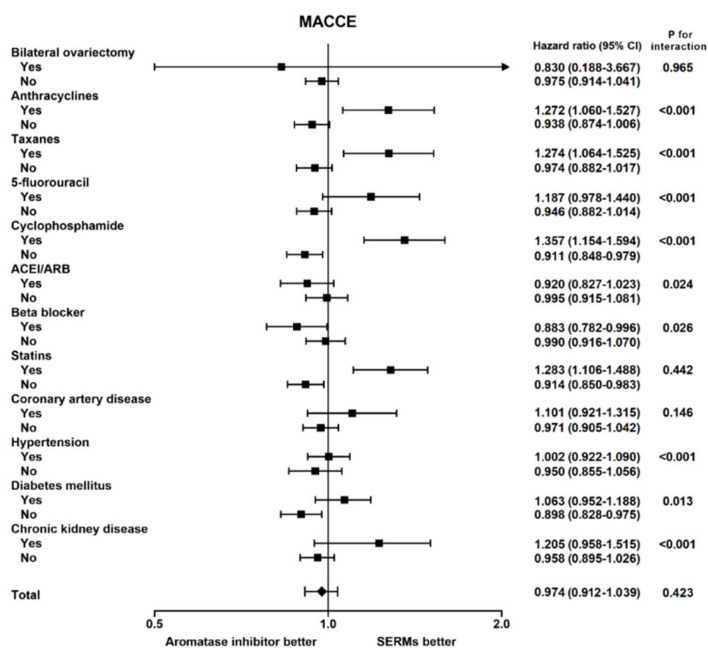
The subgroup analysis of risks of major adverse cardio-cerebral events (MACCEs) in breast cancer patients receiving either selective estrogen receptor modulators (SERMs) or Aromatase inhibitors (AIs) in the nationwide cohort.

Although AI use was associated with reduced risks of MACCEs in patients with old age or advanced cancer stage, in the subgroup analysis, we found that patients with a cumulative course of anthracycline, taxanes or cyclophosphamide exhibited a higher risk of MACCEs among AI users than SERM users (Figure 2). Additionally, among patients with cardiovascular diseases or risk factors, including CAD, DM or CKD, although insignificant, there was a trend of higher risks of MACCEs regarding AI use compared with SERM use. Conversely, patients free from cardiovascular risk factors or concomitant chemotherapy with potential cardiotoxicity were prone to having reduced risks of MACCEs using AIs instead of SERMs.

## 4. Discussion

In this Asian cohort, interestingly, we found that the risk of HF was lower in patients treated with AIs than in those treated with SERMs. When focusing on patients younger than 50 years old, the phenomenon diminished but remained significant in the older population. Similarly, in patients with advanced cancer stage, the risks of MACCEs were reduced among those who were treated with AIs. Collectively, in contrast to previous studies that showed a higher cardiovascular risk of AI use than SERM use [6,7,9], in this Asian cohort, we found that the use of AIs was associated with a lower risk of cardiovascular events than the use of SERMs among breast cancer patients in old age or at an advanced cancer stage. To our knowledge, this is the first observational study focusing on the effects of hormone therapies in Asian patients with breast cancer.

Previous studies indicated a benefit of AIs in cancer-free survival compared with tamoxifen [3,19]. The results from the Early Breast Cancer Trialists’ Collaborative Group (EBCTCG) showed that AIs reduce recurrence rates by approximately 30% compared with tamoxifen [5]. Nevertheless, subsequent meta-analyses and randomized controlled trials (RCTs) demonstrated that AIs are associated with an increased risk of ischemic events compared with tamoxifen [6,9,20]. Similarly, in the trial of Breast International Group, which focused on letrozole, a type of AI, there was a significantly increased risk of HF (0.65% vs. 0.32% in letrozole vs. tamoxifen) [21]. Khosrow-Khavar et al., reported that AI use was observed to increase the risks of HF and cardiovascular mortality compared with tamoxifen [9]. In contrast, observational studies reported inconsistent findings. Haque et al., using the Kaiser Permanente health insurance database, did not find that AIs increased risks of cardiac ischemia, stroke, HF or cardiomyopathy among women without a history of cardiovascular disease [6]. Similarly, using HealthCore Integrated Research Databases, Ligibel et al., did not observe an association between the use of AIs and AMI among women above 67 years old compared with tamoxifen use [22]. Another observational cohort study in Spain showed that AI use lowered 20% of all-cause mortality compared with tamoxifen use without increasing the risk of cardiovascular and thromboembolic events [20]. The authors even suggested that AI therapy should be the upfront option in adjuvant hormonal therapy [20]. Owing to the heterogeneity of these trials, including the study design and included populations, to date, the inconsistent findings have led to inconclusive results regarding the cardiovascular risks of AIs. Notably, the decision to initiate therapies with either AIs or SERMs mainly depends on the hormone receptor status instead of cardiovascular risk profiles [3,5]. Given that AI use is currently approved for postmenopausal women only, the aged population per se is at a higher cardiovascular risk [3,23]. Additionally, most patients under AI use concomitantly receive adjuvant chemotherapies with potential cardiotoxicity [8,24]. All abovementioned factors may contribute to unfavorable cardiovascular outcomes that were previously reported in patients receiving AIs. Most importantly, to date, there was no guideline specifically recommending the choice between AI and SERMs based on the cardiovascular risks of patients with breast cancer [12]. Consensus statement between oncologists and cardiologists mainly focused on the risk management and early detecting of cardiotoxicities [12]. 

Previous observational studies indicated an ethnic gradient in terms of the frequency of thromboembolism, and Asians have the lowest risk compared with other races [13,25,26]. Therefore, Asian women with breast cancer have unique disease characteristics and should be treated specifically. However, studies investigating the correlation between hormone therapies and cardiovascular risks in Asian women are scant and limited regarding the use of SERMs. A recently published study in Korea using a National Health Information Database focused on breast cancer patients above 55 years old and found a protective effect of tamoxifen against cardiovascular events that is absent with AIs [7]. Another study focusing on Taiwanese women with AMI also observed a 1.71 times higher risk among tamoxifen users than among nonusers [27]. However, using the Taiwan population database, Chen et al., reported no increased venous thromboembolism risk in breast cancer patients receiving adjuvant tamoxifen [28]. In contrast, our study is the first comparing the cardiovascular risks between the use of AIs and SERMs in Asian women with breast cancer and revealed a potential lower risk of MACC outcomes in AI users, especially in the aged population. Ethnicity differences should be considered as physicians choose optimal endocrine treatments for breast cancer patients.

Although AI use has been proposed to increase cardiovascular events by increasing low-density lipoprotein cholesterol levels, RCTs have not observed significant changes in cholesterol levels in patients receiving AIs [21,29]. In contrast, a plausible cardioprotective effect of tamoxifen has been reported based on its ability to lower serum lipid levels [30,31]. Nevertheless, SERM may have a different impact on lipid profiles in pre- and postmenopausal women. Although beneficial alterations of lipid metabolism were observed in premenopausal breast cancer patients treated with tamoxifen [11], in postmenopausal patients with breast cancer, marked hypertriglyceridemia was observed after tamoxifen treatment [10]. On the other hand, evidence from the Breast International Group (BIG) and Tamoxifen and Exemestane in Women With Postmenopausal Early Breast Cancer (TEAM) showed that SERMs may stimulate platelet activation and result in thromboembolic events [32,33,34]. Beyond the evidence regarding hormone therapy on thrombosis, the effect of either AIs or SERMs on myocardial function is largely unknown. Despite a proposed cardioprotective role of estrogen in females, the effects of hormone suppression or replacement therapies on cardiovascular outcomes remain controversial [35,36]. Using the UK Clinical Practice Research Datalink, it has been reported that AI use was associated with an increased risk of HF and cardiovascular death compared with tamoxifen use [9]. Conversely, in this Asian cohort, we found that HF was lower in patients treated with AIs than in those treated with SERMs. In contrast to SERMs, AIs play pivotal roles in balancing androgen and estrogen. Using a genetic model of aromatase tissue-deficient (ArKO) female mice, Bell et al. found that isolated ArKO cardiomyocytes exposed to a high Ca2+ load exhibited greater Ca2+ transient and contractile amplitudes [37]. Their findings suggested that by suppressing aromatase in females, a relative withdrawal of estrogen may support myocardial inotropes via optimized Ca2+ handling in response to stress [37]. However, whether AIs have a positive or negative impact on cardiac functions requires more investigation.

In terms of the germline genetic polymorphism, through comparing Asia Breast Cancer Consortium (ABCC) and another European Breast Cancer Association Consortium (BCAC), a meta-analysis identifying thirty-one novel breast cancer susceptibility loci unique for Asian women [38]. Additionally, the VGH-TAYLOR study is designed to understand the genetic profiling of different subtypes of breast cancer in Taiwan and define the molecular risk factors for breast cancer recurrence. Although using Genome-wide association study (GWAS), Park et al., studied genetic variants related to anthracycline-induced cardiotoxicity in early breast cancer in Korea, it remains lacking a study focusing on genetic polymorphism in the development of cardiotoxicities among breast cancer patients receiving hormone therapies including AIs and SERMs [39]. Nevertheless, genetic variability could be a possible explanation of this different effect of hormone therapies in Asian populations compared with the Western ones, but more studies are required. 

In our subgroup analysis, we found that among patients younger than 50 years old, the risks of MACCEs among AI users were similar compared with SERM users but were significantly lower in the older population. Given that, in Asia, a large portion of breast cancer patients are diagnosed before menopause, which is different from most observational studies [7,9,29], this cohort provides additional information regarding the effects of AI use on the younger population. Our findings also echo that AI use should be preserved for patients above 50 years old. In terms of cancer stage, although a meta-analysis of the Arimidex, Tamoxifen, Alone or in Combination and Breast International Group clinical trials indicated that AI use was more favorable than tamoxifen use in almost all circumstances regardless of breast cancer stage [40], in this cohort, we found that in patients with advanced breast cancer stages (stage 3–4), the risks of MACCEs were reduced most significantly among AI users compared with SERM users. However, among patients who were concomitantly or sequentially treated with chemotherapies with potential cardiotoxicities, AI use was associated with a higher risk of MACCE than SERM use. Therefore, the decision between AIs and SERMs should be tailored according to an individual’s age, cancer stage and characteristics.

Our study is not devoid of limitations. First, due to its retrospective study design, patients were not randomized to receive either SERMs or AIs. Even though the study was designed under a matching method to reduce the difference between the two groups, there was still selection bias due to patients’ menstrual status and physical conditions. To compensate for this limitation, in the subgroups analysis we divided the studied population by the age of 50 years and further investigated the impact of AI uses on cardiovascular health. Additionally, data regarding smoking, physical activity and body mass index, which may be also associated cardiovascular risks, were not available in this study. 

## 5. Conclusions

Collectively, using this nationwide Asian cohort, we observed a higher risk of MACCEs in breast cancer patients receiving SERMs than those receiving AIs, especially at ages above 50 years or having advanced cancer stages. Although the choice of hormone therapies in breast cancer patients is primarily based on their effectiveness against cancer survival, therapy-associated cardiovascular burdens remain a concern influencing long-term morbidities and quality of life. Hereby, we provide evidence regarding the role of AIs in upfront adjuvant hormonal therapy, while more investigations, especially RCTs, are mandatory to support our findings.

## Figures and Tables

**Table 1:** The baseline characteristics of breast cancer patients treated with either Aromatase inhibitors (AIs) or selective estrogen receptor modulators (SERMs) before and after inverse probability of treatment weighting (IPTW).

Caption	Inverse Probability of Treatment Weighting									
	Total		Before					After		
			SERMs		Aromatase Inhibitors		ASMD	SERMs	Aromatase Inhibitors	ASMD
	*N* = 28,458	(%)	*N* = 16,730	(%)	*N* = 11,728	(%)		*N* = Pseudo Data	*N* = Pseudo Data	
Age (mean ± SD)	54.94 ± 12.35		49.63 ± 11.55		62.53 ± 9.05		1.244	61.64 ± 25.38	59.17 ± 12.52	0.123
Age (median ± IQR)	54 ± 17		47 ± 13		61 ± 12			60 ± 31	58 ± 11	
Stage							0.515			0.101
0	1799	6.32	1454	8.69	345	2.94		5.40	5.68	
1	11,502	40.42	7558	45.18	3944	33.63		38.01	39.46	
2	11,818	41.53	6702	40.06	5116	43.62		41.32	41.75	
3	1986	6.98	753	4.50	1233	10.51		8.70	7.55	
4	1353	4.75	263	1.57	1090	9.29		6.58	5.56	
Therapies use during										
Bilateral ovariectomy	263	0.92	189	1.13	74	0.63	0.053	0.80	1.01	0.022
Anthracyclines	6215	21.84	4004	23.93	2211	18.85	0.124	17.78	19.62	0.047
Taxanes	6733	23.66	3100	18.53	3633	30.98	0.292	17.94	23.15	0.129
5-fluorouracil	5973	20.99	3907	23.35	2066	17.62	0.143	17.43	18.97	0.040
Cyclophosphamide	8071	28.36	5166	30.88	2905	24.77	0.137	23.68	25.71	0.047
CV medications										
ACEI/ARB	4197	14.75	1596	9.54	2601	22.18	0.351	19.60	18.65	0.024
Beta blocker	4063	14.28	1858	11.11	2205	18.80	0.217	18.20	17.38	0.021
Statins	2574	9.04	898	5.37	1676	14.29	0.303	11.32	11.38	0.002
Anti-platelet agents	1684	5.92	600	3.59	1084	9.24	0.232	8.20	8.00	0.008
Anti-coagulants	161	0.57	55	0.33	106	0.90	0.074	0.76	0.73	0.004
Digoxin	81	0.28	32	0.19	49	0.42	0.041	0.46	0.41	0.008
MRA	361	1.27	166	0.99	195	1.66	0.059	1.14	1.34	0.018
Comorbidities										
Coronary artery disease	1459	5.13	541	3.23	918	7.83	0.202	7.46	6.70	0.030
Peripheral artery disease	250	0.88	105	0.63	145	1.24	0.063	0.95	1.02	0.007
Hypertension	8264	29.04	3219	19.24	5045	43.02	0.531	39.26	36.29	0.061
Diabetes mellitus	4124	14.49	1500	8.97	2624	22.37	0.375	19.25	18.18	0.027
Hyperlipidemia	4723	16.60	1815	10.85	2908	24.80	0.371	20.26	20.30	0.001
Valvular heart disease	799	2.81	438	2.62	361	3.08	0.028	2.74	3.06	0.019
COPD	516	1.81	235	1.40	281	2.40	0.073	2.49	2.27	0.015
Asthma	687	2.41	361	2.16	326	2.78	0.040	2.71	2.88	0.011
Atrial fibrillation	161	0.57	57	0.34	104	0.89	0.070	0.97	0.83	0.015
Chronic kidney disease	804	2.83	287	1.72	517	4.41	0.157	3.45	3.37	0.004
ESRD	16	0.06	5	0.03	11	0.09	0.026	0.10	0.07	0.010

ASMD = absolute standardized mean difference; CV = cardiovascular; ACEI/ARB = angiotensin-converting enzyme inhibitor/Angiotensin Receptor Blocker; MRA = mineralocorticoid-receptor antagonists; ESRD = end-stage renal disease.

**Table 2:** The crude and adjusted hazard ratio (HR) of MACCE, AMI, HF and ischemic stroke in users of SERMs and AIs in NHIRD cohort.

Caption	Total *N* = 28,458	SERMs (Ref.) *N* = 16,730	Aromatase Inhibitors *N* = 11,728	Crude HR (95% CI)	*p* Value	Adjusted HR (95% CI)	*p* Value	Adjusted sHR (95% CI)	*p* Value
MACCE	1725 (6.06)	795 (4.75)	930 (7.93)	0.586 (0.552–0.622)	<0.001	0.974 (0.912–1.039)	0.423	0.970 (0.910–1.035)	0.362
HF	924 (3.25)	432 (2.58)	492 (4.20)	0.518 (0.478–0.562)	<0.001	0.884 (0.810–0.966)	0.007	0.885 (0.812–0.966)	0.006
AMI	110 (0.39)	45 (0.27)	65 (0.55)	0.422 (0.333–0.536)	<0.001	1.061 (0.814-1.382)	0.662	1.015 (0.785–1.311)	0.912
Ischemic stroke	883 (3.10)	406 (2.43)	477 (4.07)	0.655 (0.602–0.713)	<0.001	1.054 (0.961–1.156)	0.263	1.036 (0.945–1.136)	0.450

Model was adjusted for age, stage, therapies use during (bilateral ovariectomy, Anthracyclines, Taxanes, 5-fluorouracil, Cyclophosphamide), CV medication (ACEI/ARB, beta blocker, statins, anti-platelet agents, anti-coagulants, digoxin, MRA), comorbidities (coronary artery disease, peripheral artery disease, Hypertension, diabetes mellitus, hyperlipidemia, valve disease, COPD, asthma, atrial fibrillation, chronic kidney disease, ESRD). HR = hazard ratio; sHR = subdistribution hazard ratio. Abbreviations as Table 1; MACCE = major adverse cardio-cerebral events; HF = heart failure; AMI = acute myocardial infarction.

**Table 3:** The crude and adjusted hazard ratio (HR) of MACCE, AMI, HF and ischemic stroke in users of SERMs and AIs in the young (<50 y/o) and aged (≧50 y/o) population.

Young Population (<50 y/o)								
Caption	SERMs (Ref.)	Aromatase Inhibitors	Crude HR (95% CI)	*p* Value	Adjusted HR (95% CI)	*p* Value	Adjusted sHR (95% CI)	*p* Value
	*N* = 10,040	*N* = 438						
MACCE	180 (1.79)	12 (2.74)	1.384 (1.001–1.914)	0.049	0.982 (0.699–1.381)	0.919	0.976 (0.693–1.372)	0.887
HF	102 (1.02)	8 (1.83)	1.543 (1.033–2.306)	0.034	1.080 (0.713–1.634)	0.717	1.059 (0.693–1.619)	0.790
AMI	3 (0.03)	0	-		-		-	
IS	78 (0.78)	4 (0.91)	1.144 (0.658–1.988)	0.633	0.915 (0.505–1.657)	0.769	0.922 (0.529–1.607)	0.774
**Aged Population (≧50 y/o)**								
**Capation**	**SERMs (Ref.)**	**Aromatase Inhibitors**	**Crude HR (95% CI)**	***p* Value**	**Adjusted HR (95% CI)**	***p* Value**	**Adjusted sHR (95% CI)**	***p* Value**
	***N* = 6690**	***N* = 11,290**						
MACCE	615 (9.19)	918 (8.13)	0.419 (0.394–0.445)	<0.001	0.798 (0.746–0.854)	<0.001	0.820 (0.768–0.877)	<0.001
HF	330 (4.93)	484 (4.29)	0.371 (0.342–0.403)	<0.001	0.741 (0.677–0.812)	<0.001	0.776 (0.711–0.848)	<0.001
AMI	42 (0.63)	65 (0.58)	0.306 (0.241–0.388)	<0.001	0.749 (0.578–0.971)	0.029	0.760 (0.591–0.978)	0.033
IS	328 (4.90)	473 (4.19)	0.472 (0.433–0.515)	<0.001	0.877 (0.797–0.965)	0.007	0.889 (0.807–0.979)	0.017

Abbreviations as Table 1 and Table 2. ASMD = absolute standardized mean difference; CV = cardiovascular; ACEI/ARB = angiotensin-converting enzyme inhibitor/Angiotensin Receptor Blocker; MRA = mineralocorticoid-receptor antagonists; ESRD = end-stage renal disease. HR = hazard ratio; sHR = subdistribution hazard ratio. Abbreviations as Table 1; MACCE = major adverse cardio-cerebral events; HF = heart failure; AMI = acute myocardial infarction.

**Table 4:** The crude and adjusted hazard ratio (HR) of MACCE, AMI, HF and ischemic stroke in users of SERMs and AIs in breast cancer patients at different cancer stages.

Stage 0–2								
Caption	SERMs (Ref.)	Aromatase Inhibitors	Crude HR (95% CI)	*p* Value	Adjusted HR (95% CI)	*p* Value	Adjusted SHR (95% CI)	*p* Value
	*N* = 15,714	*N* = 9405						
MACCE	725 (4.61)	699 (7.43)	0.681 (0.637–0.729)	<0.001	0.930 (0.862–1.002)	0.058	0.898 (0.833–0.968)	0.005
HF	385 (2.45)	354 (3.76)	0.614 (0.559–0.674)	<0.001	0.910 (0.821–1.009)	0.074	0.878 (0.793–0.972)	0.012
AMI	41 (0.26)	39 (0.41)	0.290 (0.217–0.387)	<0.001	0.540 (0.397–0.735)	<0.001	0.529 (0.391–0.716)	<0.001
IS	380 (2.42)	389 (4.14)	0.742 (0.677–0.814)	<0.001	0.968 (0.873–1.073)	0.537	0.943 (0.850–1.047)	0.274
**Stage 3–4**								
**Caption**	**SERMs (Ref.)**	**Aromatase Inhibitors**	**Crude HR (95% CI)**	***p* Value**	**Adjusted HR (95% CI)**	***p* Value**	**Adjusted SHR (95% CI)**	***p* Value**
	***N* = 1016**	***N* = 2323**						
MACCE	70 (6.89)	231 (9.94)	0.349 (0.306–0.397)	<0.001	0.492 (0.425–0.569)	<0.001	0.589 (0.503–0.690)	<0.001
HF	47 (4.63)	138 (5.94)	0.329 (0.278–0.388)	<0.001	0.470 (0.391–0565)	<0.001	0.557 (0.453–0.684)	<0.001
AMI	4 (0.39)	26 (1.12)	1.904 (1.119–3.239)	0.0176	2.567 (1.366–4.827)	0.003	3.046 (1.657–5.601)	<0.001
IS	26 (2.56)	88 (3.79)	0.356 (0.288–0.442)	<0.001	0.434 (0.337–0.559)	<0.001	0.515 (0.383–0.693)	<0.001

Abbreviations as Table 1 and Table 2. ASMD = absolute standardized mean difference; CV = cardiovascular; ACEI/ARB = angiotensin-converting enzyme inhibitor/Angiotensin Receptor Blocker; MRA = mineralocorticoid-receptor antagonists; ESRD = end-stage renal disease. HR = hazard ratio; sHR = subdistribution hazard ratio. Abbreviations as Table 1; MACCE = major adverse cardio-cerebral events; HF = heart failure; AMI = acute myocardial infarction.

## Data Availability

Given that data is from a publicly accessible repository 3rd Party, NHIRD, data sharing is not applicable.

## Supplementary Materials

The following supporting information can be downloaded at: https://www.mdpi.com/article/10.3390/cancers14030508/s1, Figure S1: The algorithm of study design; Table S1: Lists of ICD 9 and 10 codes; Table S2: The causes of death among the studies population.
