# Supplementary material for: Risks of Aromatase Inhibitor-Related Cardiotoxicity in Patients with Breast Cancer in Asia
Source: Cancers (Basel). 2022 Jan 20;14(3):508. doi: 10.3390/cancers14030508 (PMC8833815; doi:10.3390/cancers14030508)
Supplement: Supplementary file 1 [file cancers-14-00508-s001.zip › cancers-1522342-supplementary.pdf]

Figure S1. The algorithm of study design

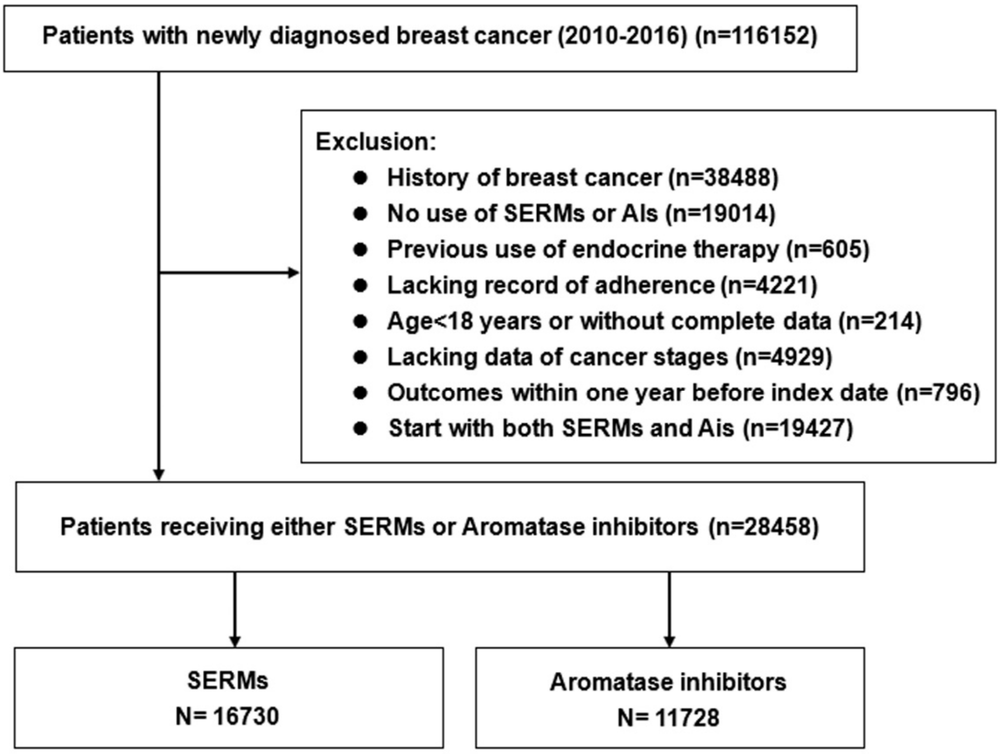

Table S1. Lists of ICD 9 and 10 codes

Disease	ICD-9 Codes	ICD-10 Codes
Breast cancer	174	C50
Outcome		
AMI	410	I21, I22, I23
CHF	428, 402.01, 402.11, 402.91, 404.01, 404.03, 404.11, 404.13, 404.91, 404.93, 785.51	I11.0, I13.0, I13.2, I42.0, I42.1, I42.2, I42.3, I42.4, I42.5, I42.6, I42.7, I42.8, I42.9, I43, I50.1, I50.20, I50.21, I50.22, I50.23, I50.30, I50.31, I50.32, I50.33, I50.40, I50.41, I50.42, I50.43, I50.9, R57.0
Ischemic stroke (including TIA)	433, 434, 435, 436	I63, I65, I66, I67.89, I67.84, G45.0, G45.1, G45.2, G45.8, G45.9, G46.0, G46.1, G46.2
Comorbidities		
Coronary artery disease (CAD)	410, 411, 412, 413, 414	I20, I21, I22, I24, I25
Peripheral artery disease (PAD)	440, 443, 444, 447.8, 447.9	I70.2-I70.9, I71, I73.9, I74.2, I74.3, I74.4, I74.5, I77.89, I77.9
Hypertension (HTN)	401, 402, 403, 404, 405	I10, I11.0, I11.9, I12.0, I12.9, I13.0, I13.2, I13.11, I15, N26.2
Diabetes mellitus (DM)	250	E08, E09, E11, E13
Hyperlipidemia	272	E78
Valve disorders	394-397, 424.0, 424.1, 424.2, 424.3	I05, I06, I07, I08, I09, I34-I37
Chronic obstructive lung disease (COPD)	491, 492, 494, 495, 496	J41, J42, J43, J44, J47, J67
Asthma	493	J45
Atrial fibrillation (AF)	427.31, 427.32	I48
Chronic kidney	580-589, 403, 404, 585, V45.1,	I12, I13, N02, N03, N04, N05,

disease	V56	N06, N07, N08, N11, N14, N17, N18, N19, N29, O10.2, O10.3, Q61, Z49, Z99.2
ESRD	585	N18.6; Z99.2

Abbreviation as listed in Table 1 and 2

Table S2.

The causes of death among the studies population

Mortality	Total N=3028	SERMs N=1078	Aromatase inhibitors N=1950	*p* value
				<0.001
Cancer death	2216 (73.18)	715 (66.33)	1501 (76.97)	<0.001
Cardiovascular death	151 (4.99)	67 (6.22)	84 (4.31)	0.023
Other causes of death	661 (21.83)	296 (27.46)	365 (18.72)	<0.001

Abbreviations as Table 1 and 2
